# Development of PMMA composites with tungsten trioxide for improved gamma radiation shielding in microsatellites

**DOI:** 10.1038/s41598-025-94120-z

**Published:** 2025-04-17

**Authors:** Nourhan Hesham, Hosnia M. Abu-Zeid, A. Nada, Ibrahim S. Yahia, Mai S. A. Hussien, H. M. Diab, Mohammed AbuBakr Ali, Ahmed T. Mosleh, Dalia Elfiky

**Affiliations:** 1 National Authority for Remote Sensing & Space Sciences, Space Environment, Cairo, 11769 Egypt; 2 Present Address: Physics Department, Faculty of Women for Arts, Science and Education, Ain Shams University, Cairo, 11765 Egypt; 3 Nanoscience Laboratory for Environmental and Bio‑medical Applications (NLEBA) Green Research Laboratory (GRL), Chemistry, Faculty of Education, Ain Shams University, Cairo, 11757 Egypt; 4 Egyptian Atomic Energy Authority, Radiation Control, Cairo, 11765 Egypt

**Keywords:** PMMA/WO_3_ composite, Gamma rays, X-ray diffraction (XRD) analysis, Differential scanning calorimetry (DSC), Point gamma sources, Space physics, Chemistry, Materials science, Nanoscience and technology, Physics

## Abstract

Satellites are exposed to various types of radiation and extreme temperatures in space, which can lead to damage and malfunctioning of critical components. Therefore, the use of an efficient shielding system is essential for ensuring the longevity and performance of satellites. In the ever-evolving landscape of space exploration, the demand for resilient and efficient materials to safeguard the delicate internal components of microsatellites has never been more critical. This work investigated the gamma-ray shielding performance, and the physical and mechanical properties of poly (methyl methacrylate) (PMMA) composites embedded with 0–50 wt% tungsten trioxide (WO_3_). The results showed that the addition of WO_3_ had significantly improved the gamma shielding ability of PMMA composites. Linear attenuation coefficient and half-value layer were examined using three gamma sources (Cs-137, Ba-133, and Co-60).

## Introduction

Space presents ongoing challenges for satellites, even with significant technological advancements over the last thirty years. Unlike Earth, space lacks natural protection, exposing satellites to a spectrum of high-energy electromagnetic radiation, including ultraviolet (UV) radiation, X-rays, and gamma rays. These forms of radiation pose significant risks to satellite integrity and functionality. Gamma rays, possessing wavelengths less than 0.01 nm and energies exceeding 100 keV, are the most penetrating and energetic, capable of traversing substantial shielding and causing deep-seated damage to electronic systems. Among these, gamma rays are particularly concerned due to their high penetration power, necessitating effective shielding strategies to protect satellite electronics and maintain mission integrity^1^.

Traditional shielding materials such as lead, copper, and stainless steel have been employed to mitigate the effects of space radiation. Lead, for instance, has been widely used due to its high density and atomic number, which provide substantial attenuation of gamma rays. However, the significant mass of these materials poses challenges for space applications, where weight is a critical constraint due to launch costs and payload limitations. Additionally, environmental and health concerns associated with materials like lead have prompted the search for alternative solutions. The rigidity of these traditional materials also limits their applicability in shielding complex or delicate satellite components that may require more adaptable materials^2,3^.

In response to these challenges, research has increasingly focused on polymer-based composites incorporating high atomic number (high-Z) fillers. Polymers such as poly(methyl methacrylate) (PMMA) offer advantages including low density, mechanical flexibility, and ease of fabrication. When combined with high-Z materials like tungsten oxide (WO_3_), these composites can achieve enhanced radiation shielding properties while maintaining a reduced overall weight. WO_3_ is particularly promising due to its high atomic number and density, which contribute to effective attenuation of high-energy photons, including gamma rays. Furthermore, WO_3_ exhibits excellent thermal stability and chemical durability, essential for the extreme conditions encountered in space environments^3,4^.

Recent studies underscore the promise of high-Z material composites for radiation shielding. El-Khatib et al.^5^ demonstrated the effectiveness of PMMA reinforced with tungsten carbide (WC), achieving superior gamma attenuation and mechanical performance compared to traditional materials. Similarly, research by Aghaz et al.^6^ and Elsafi et al.^7^ highlighted the benefits of incorporating nano-structured WO_3_ into polymeric matrices, improving photon attenuation and shielding effectiveness^8^. Additional work by Hussan et al.^9^ and Alorain et al.^10^ further supported WO_3_’s potential, showing its ability to enhance shielding performance in various composite systems, including silicon epoxy resins and polydimethylsiloxane^11,12^. These studies primarily focused on varying the thickness of the shielding samples to evaluate the linear attenuation coefficient (LAC).

In evaluating the effectiveness of radiation shielding materials, several key parameters are considered alongside the mass attenuation coefficient (MAC). The half-value layer (HVL) represents the thickness of a material required to reduce the intensity of incident radiation by half. Similarly, the tenth-value layer (TVL) is the thickness needed to attenuate the radiation to one-tenth of its original intensity. The mean free path (MFP) denotes the average distance a photon travels within a material before interacting with it. These parameters are crucial for designing and assessing shielding materials, as they provide insights into the material’s capacity to attenuate radiation effectively. Materials with lower HVL, TVL, and MFP values are generally more efficient at shielding against radiation, as they require less thickness to achieve the desired attenuation.

In this study, we investigate the effect of varying WO_3_ filler concentrations on the MAC, HVL, TVL, and MFP of PMMA/WO_3_ composites. By systematically adjusting the WO_3_ content, we aim to identify an optimal composition that balances effective gamma radiation attenuation with the lightweight and mechanical properties required for satellite applications. This approach seeks to provide a more efficient and practical solution for radiation shielding in space, addressing the critical need for materials that offer protection without adding prohibitive weight. The novelty of this work lies in its focus on filler concentration to optimize radiation shielding performance, diverging from traditional methods that rely on increasing material thickness. By enhancing our understanding of how WO_3_ concentration affects these key shielding parameters in PMMA composites, this research contributes to the development of advanced materials tailored for the stringent requirements of space missions.

## Theoretical background

This revised theoretical background focuses specifically on the interaction of gamma radiation with PMMA/WO_3_ nano-composites for radiation shielding applications in microsatellites.

## Gamma radiation interaction

### Photon interaction

Unlike charged particles like protons and electrons, gamma rays interact with matter primarily through three mechanisms:

- Photoelectric effect: The gamma ray is absorbed by an atom, causing the ejection of an electron. This effect is dominant for low-energy gamma rays and high-atomic-number materials.
- Compton scattering: The gamma ray collides with an electron, transferring some of its energy to the electron and changing its direction. This effect is dominant for intermediate-energy gamma rays.
- Pair production: The gamma ray interacts with the nucleus of an atom, creating an electron–positron pair. This effect is dominant for high-energy gamma rays.

### Photon attenuation

The attenuation of gamma rays through a material is described by the exponential decay law shown in Eq. (1):

1 $${\text{I }} = {\text{ I}}_{0} {\text{exp}}\left( { -\upmu {\text{x}}} \right)$$

where I is the transmitted intensity. I_0_ is the initial intensity. μ is the mass attenuation coefficient. x is the thickness of the material.

The mass attenuation coefficient depends on the energy of the gamma rays and the composition of the material. It can be estimated using theoretical models or obtained from experimental data^1^.

#### Shielding effectiveness

The effectiveness of a material as a gamma ray shield depends on its ability to attenuate the radiation. This can be quantified by the following parameters:

- Half-value layer (HVL): The thickness of the material required to reduce the intensity of the gamma rays by half as shown in Eq. (2).
  2 $${\text{HVL }} = {\text{ ln}}\left( {2} \right) \, / \,\upmu$$
- Tenth-value layer (TVL): The thickness of the material required to reduce the intensity of the gamma rays by a factor of ten as shown in Eq. (3).
  3 $${\text{TVL }} = {\text{ ln}}\left( {{1}0} \right) \, / \,\upmu$$
- The mean free path (MFP) represents the average distance between two successive interactions between gamma rays and a sample as shown in Eq. (4).
  4 $${\text{MFP }} = { 1}/\upmu$$

Materials with high atomic numbers and high densities generally have better gamma ray shielding properties than materials with low atomic numbers and low densities^13^.

## Results

### Shield effectiveness

**Fig. 1:**
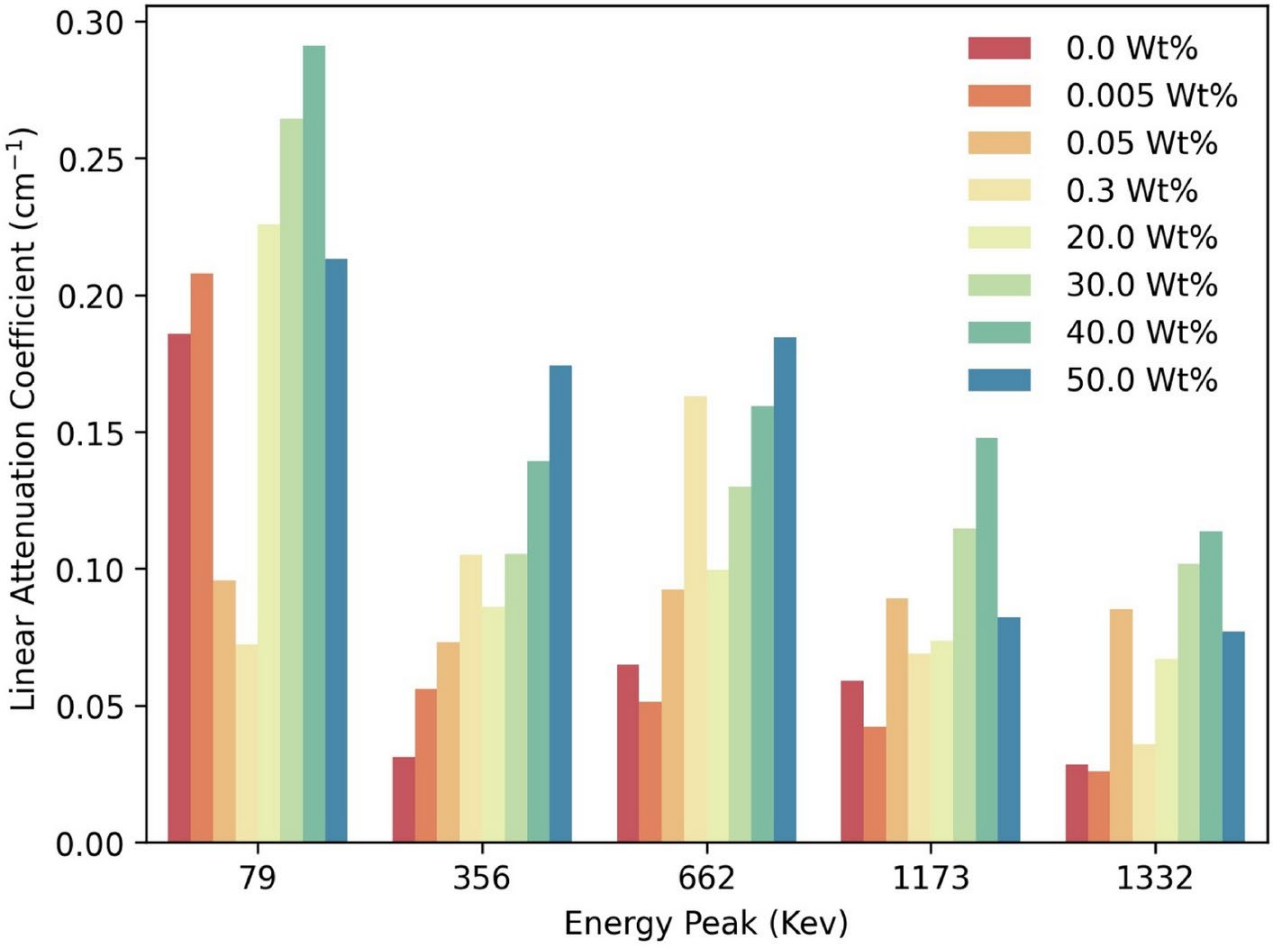
The change of linear attenuation coefficient with different gamma ray energies for different WO_3_ concentrations in the composite.

Figure 1 the linear attenuation coefficient (LAC) of PMMA/WO₃ composites demonstrates a clear enhancement with increasing WO_3_ concentration, particularly at photon energies below 1000 keV. This improvement is primarily due to the photoelectric effect, where gamma photons are completely absorbed through energy transfer to electrons in the shielding material. Tungsten oxide, with its high atomic number (Z = 74), increases the probability of these photoelectric interactions, making it an effective material for gamma-ray attenuation.

At a concentration of 40%, the composite achieves a suitable balance, with an LAC of approximately 0.15 cm^−1^ at 662 keV, making it practical for space shielding applications.

As photon energy increases, the influence of the photoelectric effect decreases, and Compton scattering becomes the dominant interaction mechanism. This transition results in a reduced LAC at higher photon energies, such as 1173 keV and 1332 keV, regardless of WO_3_ concentration. While adding WO_3_ to PMMA enhances gamma-ray shielding at lower energies, the attenuation efficiency naturally diminishes at higher energies due to the reduced sensitivity of Compton scattering to the material’s atomic number.

It is important to note that at WO_3_ concentrations exceeding 40%, such as 50%, particle agglomeration may occur, leading to non-uniformities within the composite. These non-uniformities can negatively impact the material’s mechanical integrity and overall performance. Thus, optimizing the WO_3_ content is critical to achieving a composite that offers effective radiation shielding while maintaining structural integrity and minimizing weight—key considerations for space applications.

This analysis demonstrates that the composite’s performance at 40% WO_3_ concentration, particularly at 662 keV, aligns well with the requirements for lightweight and efficient shielding materials for use in microsatellites and other space applications^13^.

**Fig. 2:**
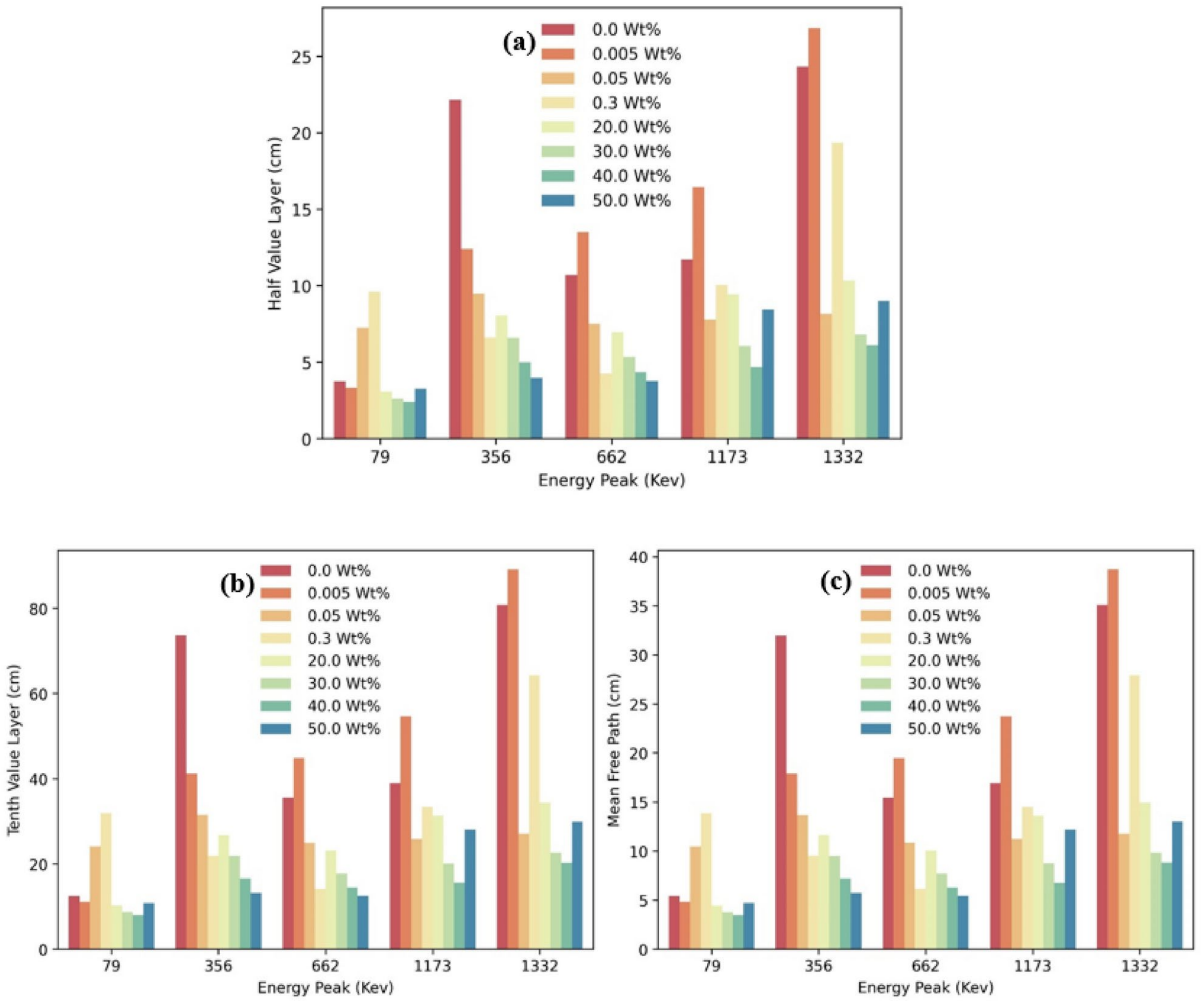
(**a**–**c**) HVL, TVL, and MFP of PMMA composites with different loadings of WO_3_ particles.

Figure 2a illustrates the half-value layer (HVL), which is the thickness of a material required to reduce the intensity of incoming radiation by half. HVL is crucial for assessing the radiation shielding capabilities of polymer composites like PMMA/WO_3_. When more WO_3_ is added to PMMA, the HVL usually decreases, indicating better radiation shielding. This is because tungsten, with its high atomic number, is more effective at scattering and absorbing radiation. However, at a 50% concentration of WO_3_, the HVL starts to increase again. This suggests complex interactions between WO_3_ nanoparticles and the polymer. Understanding these interactions is important for optimizing radiation shielding materials for specific applications, balancing better attenuation with practical material composition.

The findings highlight that the 50% WO_3_ concentration faces challenges such as particle agglomeration, which leads to non-uniformity and reduced mechanical integrity. However, at 40% WO_3_ concentration, the composite achieves a significant balance, with a linear attenuation coefficient (LAC) of approximately 0.15 cm^−1^ at 662 keV. This makes it suitable for space applications due to its efficiency and structural integrity.

At higher photon energies, where Compton scattering dominates, the attenuation performance naturally diminishes for all concentrations. Nevertheless, 40% WO_3_ offers optimal performance without the drawbacks observed at higher filler content, such as agglomeration or increased HVL. This explanation aligns the results with the conclusion, identifying 40% as the optimal concentration for most energy ranges (particularly at 662 keV) while noting the specific use cases for 50% WO_3_ within the 100–1000 keV range.

Besides the half-value layer (HVL), two other important factors in radiation shielding are the tenth-value layer (TVL) and the mean free path (MFP) Fig. 2b,c, respectively. The TVL measures the thickness of a material needed to reduce radiation to one-tenth of its original intensity. The MFP represents the average distance a radiation photon travels before significantly interacting with the material.

Changes in the concentration of WO_3_ inside the PMMA/WO_3_ composite can affect these values. Both the TVL and MFP usually decrease with increasing WO_3_ content, suggesting increased radiation attenuation capabilities. Because of its higher atomic number, tungsten is a better material for shielding since it can absorb and scatter radiation more effectively.

The increase in the HVL at 50% WO_3_ concentration could also impact the TVL and MFP. This behaviour suggests that the interactions between WO_3_ nanoparticles and the polymer matrix change at this concentration, affecting the material’s overall ability to block radiation.

To maximize radiation shielding effectiveness, it is critical to understand how the TVL and MFP change in the PMMA/WO_3_ composite. Balancing the specific needs of the application, the material’s structure, and the WO_3_ concentration is essential. Further research and testing are needed to understand these behaviours better and to optimize the composite for improved radiation attenuation in practical applications.

50% concentration of WO_3_ in PMMA becomes less effective at energy levels above 1000 keV because it can cause secondary emissions, which reduce the material’s overall attenuation efficiency. In contrast, a 40% concentration is more optimal in this energy range, as it better balances radiation shielding without leading to these secondary effects, maintaining higher attenuation performance.

50% concentration of WO_3_ is also less effective for gamma rays with energies below 100 keV. This is because the weaker energy levels combined with the tendency of the filler to clump together (agglomeration) reduce the material’s ability to attenuate the radiation. In contrast, the 40% concentration avoids this issue, leading to better overall performance in this energy range.

WO_3_ at 50% concentration works best and is most effective for gamma radiation shielding in the energy range of 100–1000 keV. This is because, in comparison to other concentrations, it achieves the ideal and optimal balance between the amount of additive and the energy of the incoming gamma rays (absorption), enabling the material to maximize its radiation attenuation properties and offer superior protection.

### Structure analysis

**Fig. 3:**
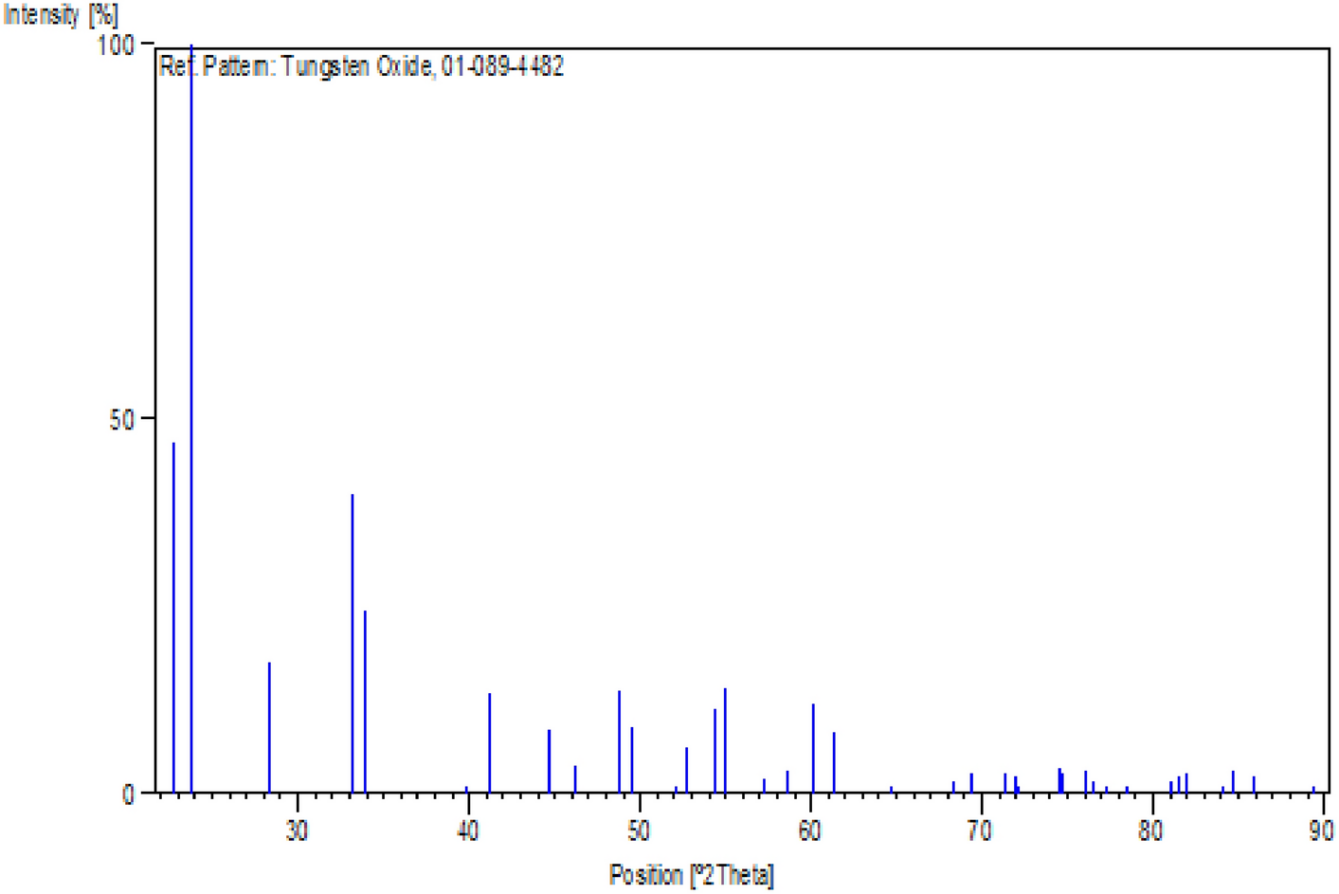
The peak of reference card of WO_3_ powder from XRD analysis from program.

Figure 3 shows how the reference card helps us tell apart the expected peaks from any new ones when we add new materials or change the experiment. By comparing the XRD patterns, we can clearly see the differences between the composite material and the WO_3_ component.

**Fig. 4:**
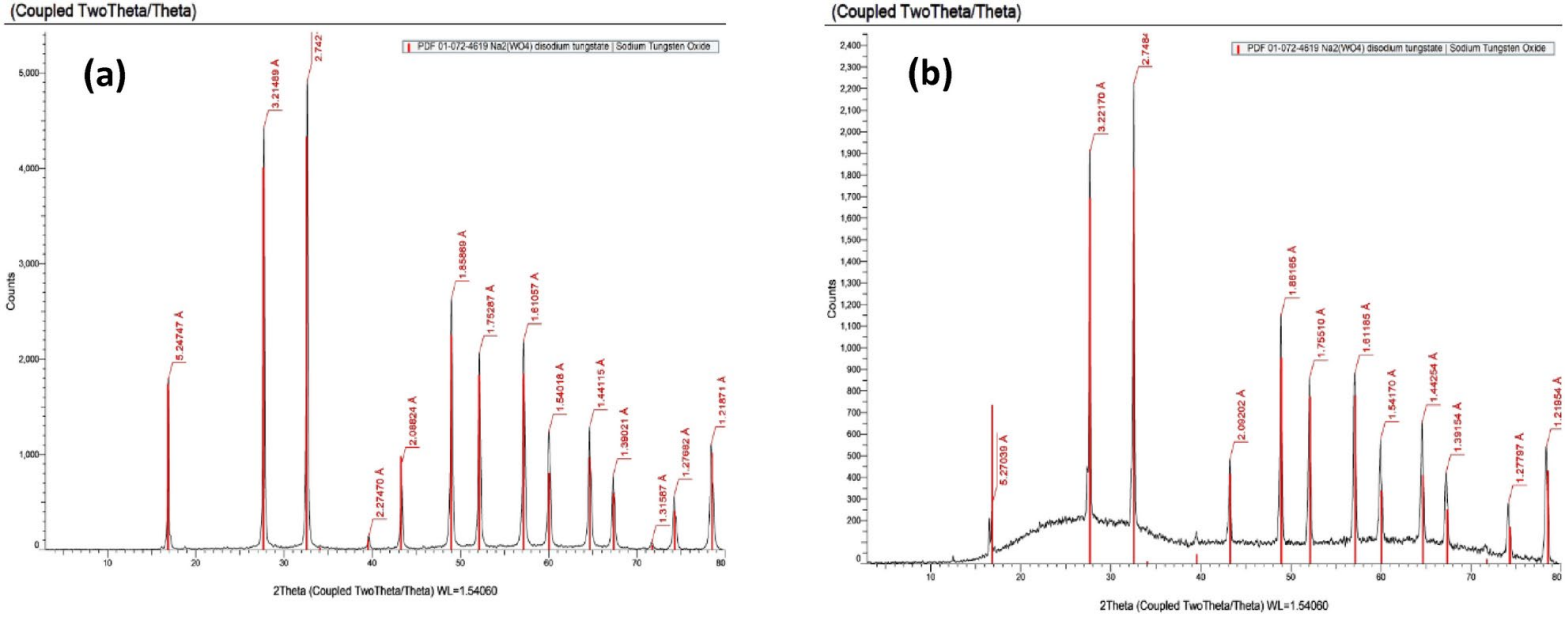
(**a**, **b**) The peaks of WO_3_ powder from XRD analysis (**a**) before and (**b**) after radiation.

**Fig. 5:**
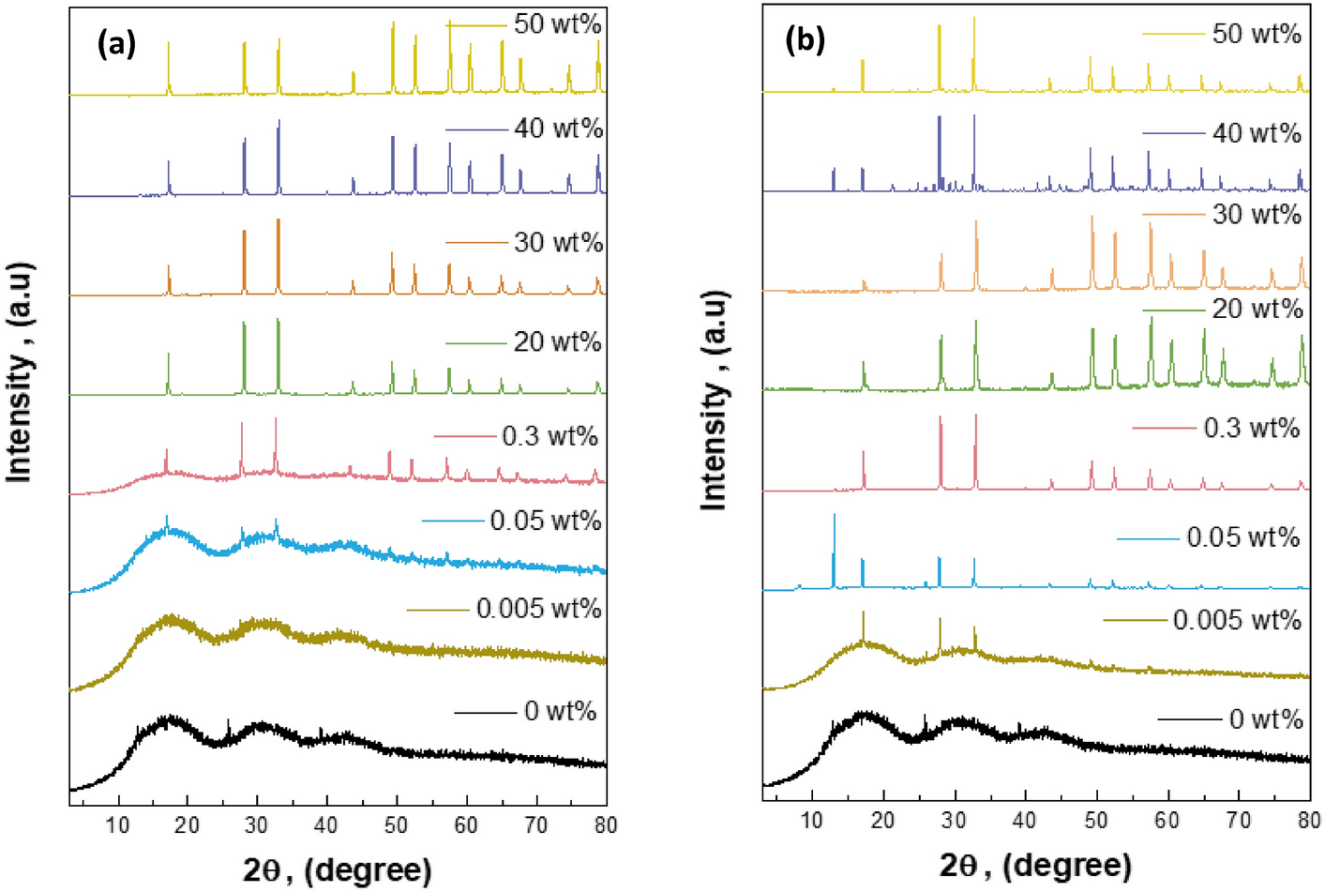
(**a**, **b**) XRD analysis of PMMA/WO_3_ composites with different concentration of WO_3_ (**a**) before and (**b**) after radiation.

The reference card ensures accurate XRD analysis, making it a useful tool for understanding the unique properties and crystalline structures of composite materials. Figure 4a,b show that the XRD study of the pure sample reveals only minor changes before irradiation (Fig. 4a) and after irradiation (Fig. 4b), indicating a stable crystalline structure. However, distinct WO_3_ peaks appear gradually as more WO_3_ is added. These peaks become more noticeable after radiation exposure. This happens because the radiation interacts with the composite material, causing the formation of WO_3_ peaks. Figure 5a,b show the XRD patterns of the PMMA/WO_3_ composite at different concentrations, with Fig. 5a representing the sample before irradiation and Fig. 5b after irradiation. The diffractogram of pure PMMA shows an intense peak at 2θ = 17.30° and a weak peak at 2θ = 29.81°. It is observed that with the increase in the concentration of WO_3_ nanocomposite, the intensity of the characteristic peak at 17.30° decreases and becomes broader. The intermolecular interaction between PMMA chains through hydrogen bonding is attributed to the semi-crystalline nature of PMMA, and it is revealed that as the concentration of the WO_3_ increases, these bonds tend to weaken^14^. The interaction between the nanofiller and PMMA chains increases whereas the interaction between the PMMA chains with each other decreases with the improvement in the amorphousity of the PMMA nanocomposites^15^. At 0.05 wt% of WO_3_ in PMMA matrix, the peak of WO_3_, that appear and matched with card no. (01-089-4482)^16^. Before XRD, use the reference card to typical the materials in it and ensure that they have the same peaks of standard, a reference card helps accurately identify and analyze WO_3_ phases using XRD. This is very useful for studying PMMA/WO_3_ composites. The results indicate that radiation causes changes in the polymer matrix, making previously obscured WO_3_ crystalline phases visible in Fig. 5b. As more WO_3_ is added, it gradually integrates into the composite, shown by its step-by-step appearance with increasing concentration. The new crystalline patterns after radiation highlight the material’s changing nature. The changes in the crystalline structure due to gamma radiation are detectable through XRD patterns, which reveal variations in crystallite size and phase composition. These structural modifications are indicative of the material’s response to radiation exposure^17^. Upon exposure to ionizing radiation, polymers can undergo chain scission, leading to the breakdown of their molecular chains. This degradation process can result in the formation of free radicals and smaller molecular fragments. In the context of a PMMA/WO_3_ composite, such radiation-induced degradation of the PMMA matrix may facilitate the reorganization or crystallization of WO_3_ particles within the composite. XRD is a valuable tool for observing these structural changes caused by adding WO_3_ and the effect of radiation on the composite’s crystalline properties.

**Fig. 6:**
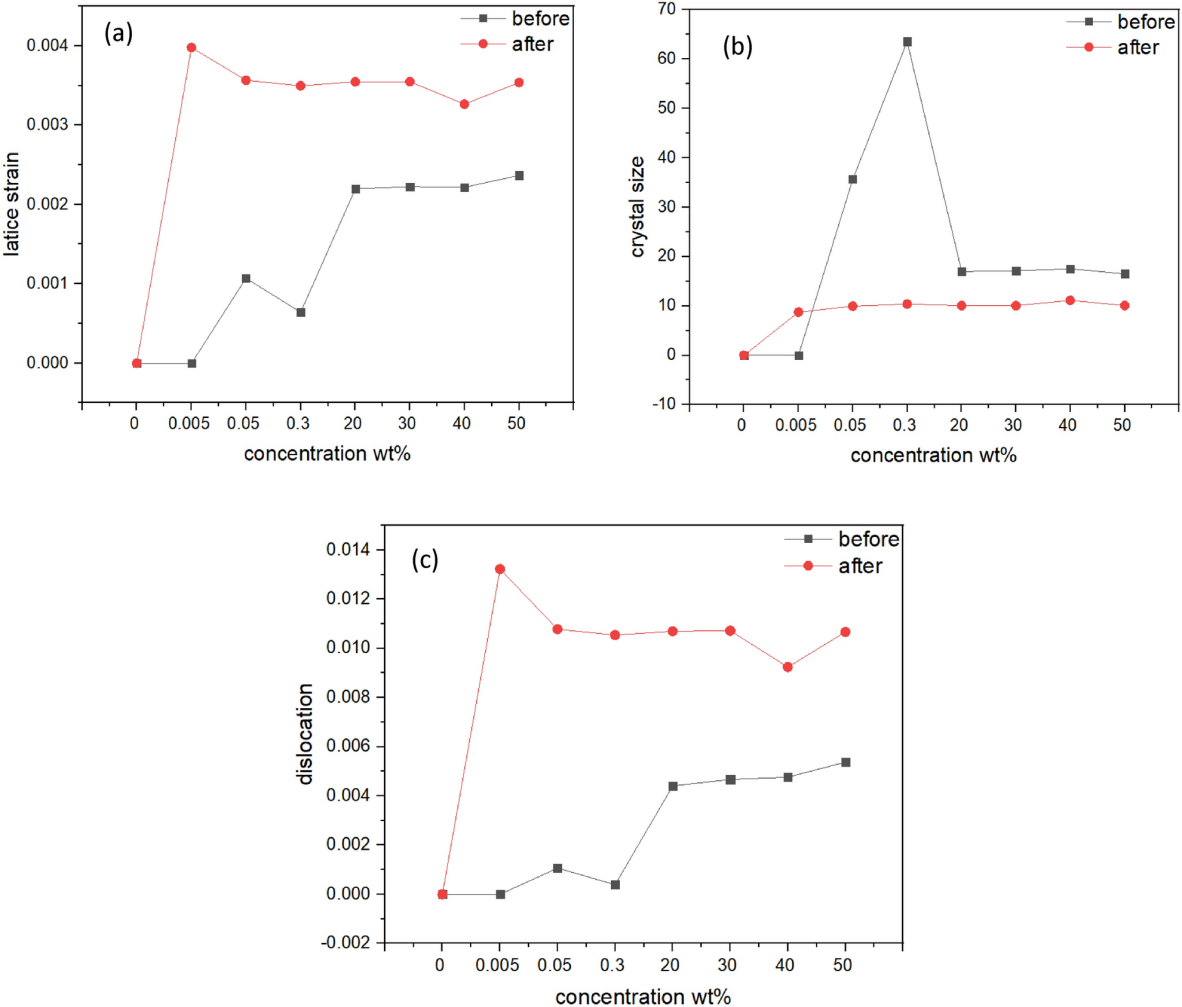
(**a**) The crystal size, (**b**) dislocation and (**c**) lattice strain for different concentrations of PMMA/WO_3_.

The crystal size, dislocation properties, and crystal strain of PMMA/WO_3_ nanocomposites show significant changes with varying concentrations of WO_3_ and exposure to gamma radiation, as seen in Table 1 and Fig. 6a–c. Before exposure to radiation, increasing the concentration of WO_3_ in the composite leads to larger crystal sizes as shown in Fig. 6b, peaking at 0.3 wt% before stabilizing or decreasing slightly. This suggests that 0.3 wt% WO_3_ is optimal for maximum crystal growth or arrangement in the PMMA matrix. However, when the composite is exposed to gamma radiation, the crystal size decreases, implying that the radiation impacts the material’s structure by altering the arrangement or stability of the crystals. The relationship between WO_3_ concentration and radiation is complex and needs more investigation to understand how these factors interact and affect the nanocomposite’s structure. Additionally, as shown in Fig. 6c the number of dislocations in the material increases with higher WO_3_ content before irradiation, indicating a connection between WO_3_ and dislocation formation in the PMMA. Exposure to gamma radiation further raises the dislocation density, suggesting that radiation significantly influences the dislocation structure, potentially affecting the material’s strength. As shown in Fig. 6a the crystal strain also follows a clear trend: it increases with higher WO_3_ concentrations and rises further under gamma radiation, indicating structural changes within the composite. Understanding these behaviours and mechanisms is essential for determining how the nanocomposite’s properties respond to various conditions, particularly under radiation.

**Table 1:** The change in crystal size, dislocation and lattice strain for PMMA/WO_3_ before and after gamma irradiation.

Concentration (PMMA/WO_3_)	Crystal size		Dislocation		Lattice strain	
	Before	After	Before	After	Before	After
Pure PMMA	–	–	–	–	–	–
0.005 wt%	–	8.739508	–	0.01323	–	0.00398
0.05 wt%	35.68031	9.938783	0.001069	0.01078	0.001072	0.003566
0.3 wt%	63.55682	10.3765	0.000394	0.01054	0.000642	0.003497
20 wt%	16.96186	10.05034	0.004412	0.0107	0.002198	0.003548
30 wt%	17.10858	10.05518	0.004673	0.01072	0.002226	0.003549
40 wt%	17.49582	11.11551	0.004762	0.00924	0.002218	0.003265
50 wt%	16.49875	10.07858	0.005381	0.01067	0.002368	0.00354

### Thermal characterization

From Table 2, it is clear that, the glass transition temperature (Tg) of WO_3_ changed with its concentration in the PMMA. At moderate WO_3_ concentrations (0.005–0.3 weight percent), Tg increased noticeably compared to pure PMMA, reaching around 120.8 °C and 122.7 °C before and after irradiation, respectively. However, at higher concentrations (20–50 weight percent), Tg was decreased again. This reduction is likely due to interactions and changes in the composite’s structure.

**Table 2:** Tm and Tg of composite PMMA/WO_3_ before and after irradiation.

Concentration (%)	Before		After	
	Tg	Tm	Tg	Tm
0	111	376	114	375.8
0.005	118.6	379	120.6	379.7
0.05	120.7	377	122.6	377.7
0.3	120.8	377	122.7	377
20	114	377	115.7	377
30	112	376	114	376.8
40	107	375	111.6	373.4
50	105	373	108	373.3

After radiation exposure, notable changes occurred, in both Tg and Tm which decreased at higher WO_3_ concentrations (40 and 50 weight percent) Table 2. However, Tm showed fluctuations, with occasional increases and decreases. These changes are related to the internal structure of the composite and the preparation of the PMMA/WO_3_ samples.

**Fig. 7:**
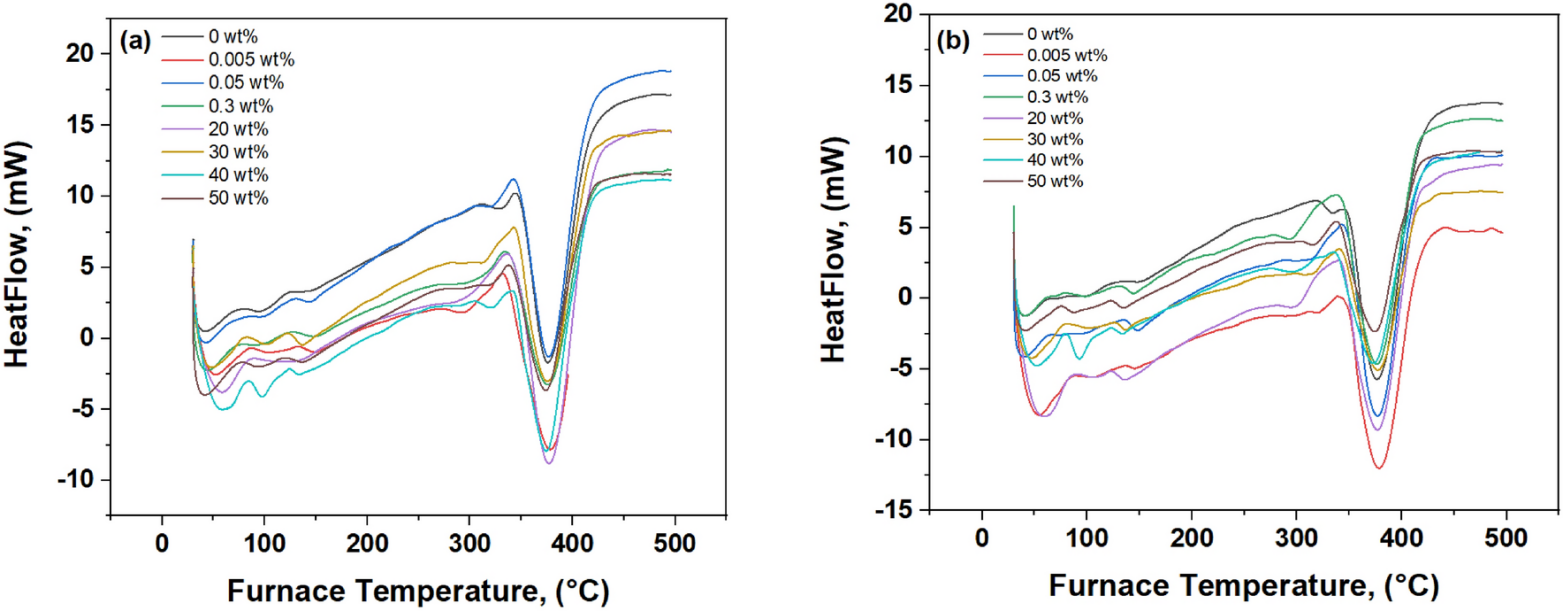
(**a**, **b**) DSC thermograph (**a**) before and (**b**) after gamma irradiation.

Figure 7a,b, The DSC thermogram provides insights into how PMMA and WO_3_ interact at different concentrations. Changes in the shape of peaks, heat flow, and baseline can give clues about how well the materials stick together, the structure of the composite, and any crystalline phases present. To fully understand the thermal behaviour and structure of PMMA/WO_3_ composites, DSC results should be combined with other techniques like TGA, FTIR, and XRD.

**Fig. 8:**
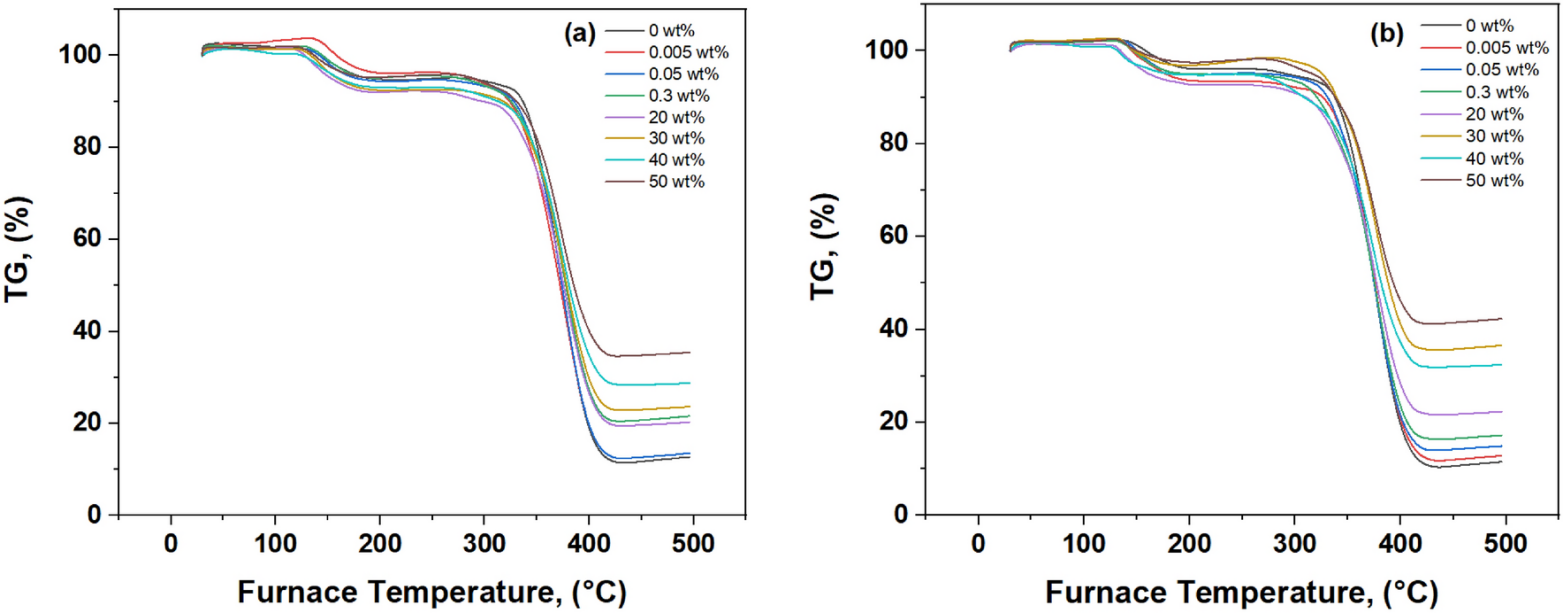
(**a**, **b**) TGA thermograph (**a**) before and (**b**)after gamma irradiation.

Figure 8a,b shows that pure PMMA (0% WO_3_) typically starts to degrade around 350 °C, with the main loss of mass occurring between 340 and 400 °C due to the breaking of polymer chains. When WO_3_ is added at concentrations of 0.005–0.3 weight percent, the start of degradation may either remain the same as pure PMMA or experience a slight delay. This change is due to potential interactions between PMMA and WO_3_.

At higher WO_3_ concentrations (20–50 weight percent), the onset temperature of degradation may shift to higher values. This is likely because the thermally stable WO_3_ particles act as heat sinks, delaying the initial breakdown of PMMA chains.

While radiation usually breaks polymer chains, it can also cause a surprising effect: cross-linking. This process forms covalent bonds between nearby polymer chains, creating a stronger network. In PMMA/WO_3_ composites, cross-linking might happen within the PMMA or between PMMA and WO_3_ at their interface. The effects of this cross-linking are quite interesting. Radiation can both break polymer chains, speeding up degradation, and create cross-links, which improve thermal stability. The overall effect depends on the balance between these two processes. At lower radiation doses, cross-linking may be more dominant, leading to a higher onset temperature and slower degradation rate.

### FTIR analysis

**Fig. 9:**
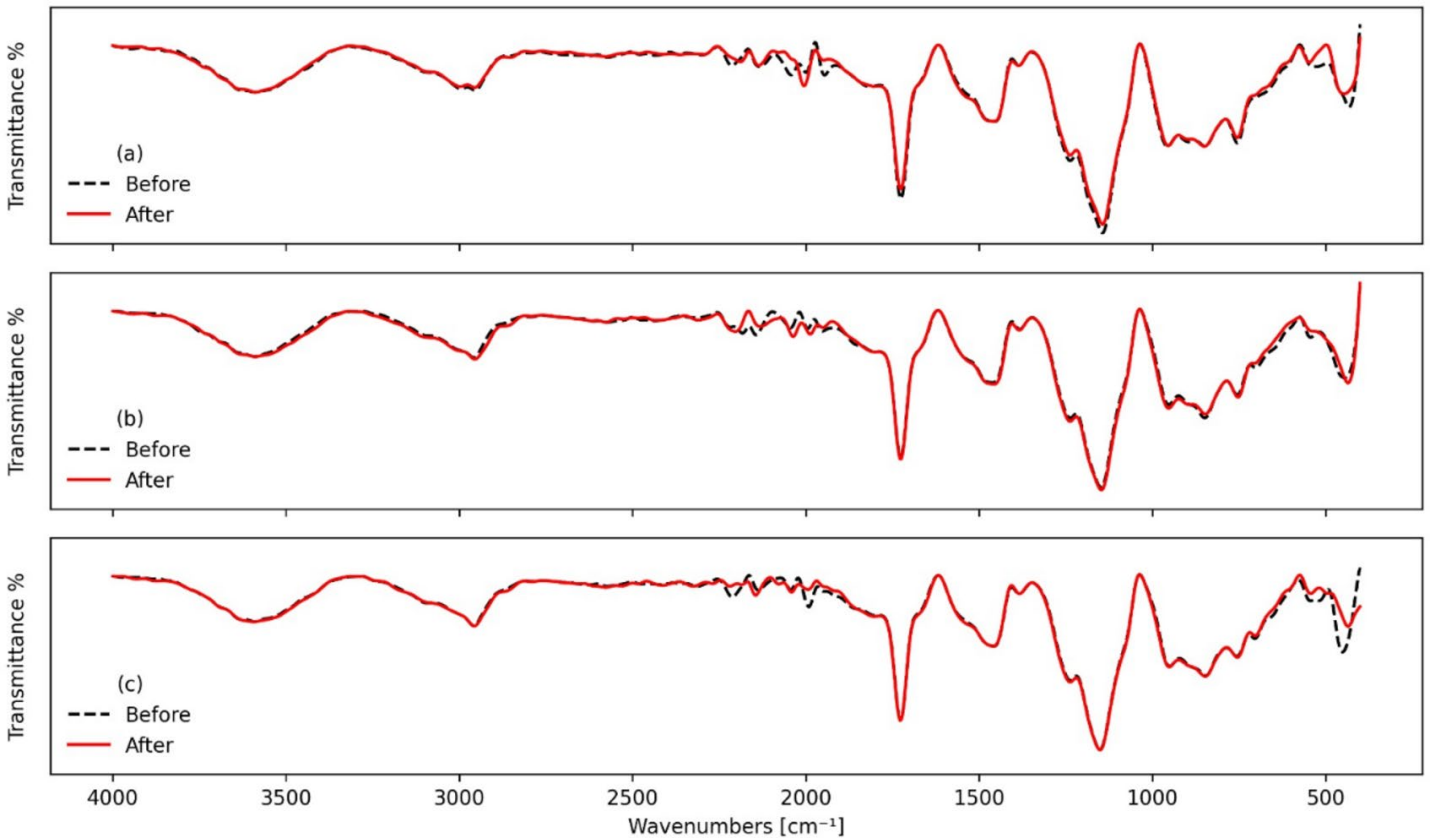
FTIR (**a**) 0 wt%, (**b**) 40 wt%, and (**c**) 50 wt% before and after gamma irradiation.

In Fig. 9, the FTIR analysis of PMMA and PMMA/WO_3_ composites at different WO_3_ concentrations (0% Fig. 9a, 40% Fig. 9b, and 50% Fig. 9c) reveals varying structural changes after gamma radiation. For pure PMMA, radiation causes a slight shift in the carbonyl (C=O) peak, indicating bond weakening and the possible formation of new groups. Broadening in certain regions and changes in peak intensities suggest structural modifications, such as chain scission, crosslinking, or oxidation that introduces groups like hydroxyl (OH) or carboxyl (COOH). In the 40% WO_3_ composite, WO_3_ adds new peaks (notably below 1000 cm^−1^) and appears to modify PMMA’s radiation response, potentially acting as a shield or sensitizer. This composite shows similar peak shifts, broadening, and signs of PMMA degradation but with potentially reduced severity due to WO_3_’s presence. At 50% WO_3_, WO_3_’s influence is more pronounced, masking some PMMA peaks and possibly providing more substantial shielding against radiation. While PMMA still undergoes some degradation, the high WO_3_ content seems to alter how radiation affects the composite, introducing new interactions that could lead to compound formation or WO_3_ structural changes. Across all samples, radiation induces complex structural modifications, with higher WO_3_ concentrations potentially mitigating some radiation damage to PMMA while introducing new interactions within the composite.

### FESEM analysis

**Fig. 10:**
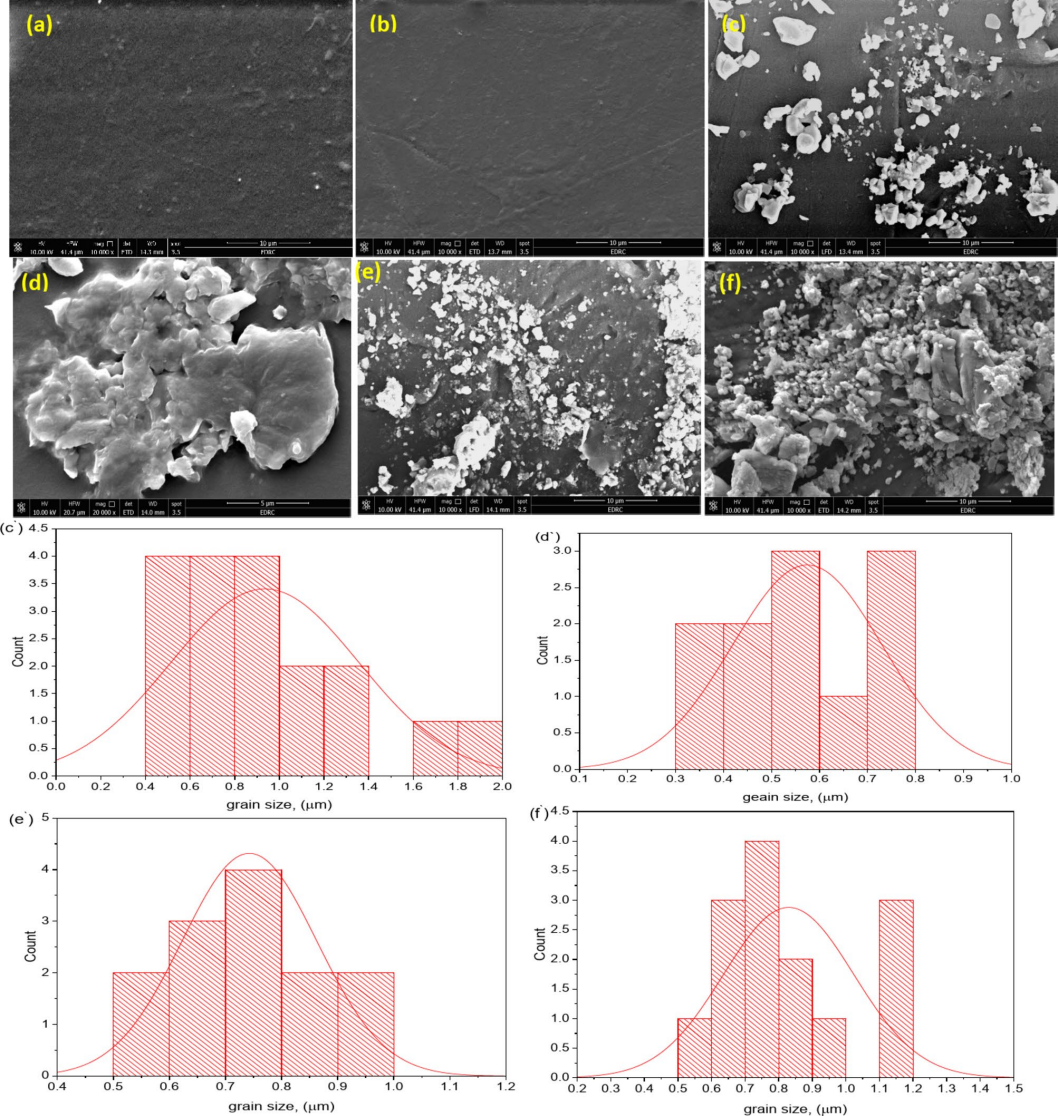
SEM image of the pure polymer PMMA (**a**, **b**), 40% (**c**, **d**), and 50% (**e**, **f**) of WO_3_ before and after irradiation, respectively, 40% (**c**′, **e**′), and 50% (**d**′, **f**′) average grain size.

The morphological structure of the PMMA/WO_3_ composite was analyzed using SEM to study the distribution and dispersion of WO_3_ nanoparticles (NPs) within the polymer matrix, which is critical for enhancing the material’s properties. Uniform dispersion of WO_3_ NPs is preferred to prevent the formation of stress concentrators or crack initiators in the PMMA film. Figure 10a shows the SEM image of pure PMMA, revealing a smooth, uniform surface with no visible defects, indicating high-quality synthesis. Even after gamma irradiation, as seen in Fig. 10b, the surface remains smooth, demonstrating that pure PMMA resists radiation damage at this scale. Figure 10a–f depict PMMA/WO_3_ composites with varying WO_3_ concentrations (0–50 wt%). At lower concentrations, the WO_3_ nanoparticles are dispersed evenly, as shown in Fig. 10c. The average particle size of the WO_3_ in the composite was approximately 100–150 nm, based on image analysis of SEM micrographs. However, at higher concentrations (e.g., 40–50 wt%), agglomeration is observed, resulting in clusters of WO_3_ particles. These clusters exhibit varied particle sizes, with average cluster sizes ranging from 800 nm to 1 µm as shown in Fig. 10c′,e′), SEM image in Fig. 10e shows the PMMA matrix containing densely packed 50% WO_3_ particles before gamma irradiation, displaying varied particle sizes and shapes, which suggests a heterogeneous distribution. The presence of tungsten, a high atomic number element, likely enhances the composite’s gamma shielding properties. The WO_3_ particles appear in high concentration and are only partially embedded in the PMMA. After irradiation, as depicted in SEM images (Figs. 10d,f) reveal a reduction in cluster size and a more uniform distribution of WO_3_ particles. The compaction or fragmentation of WO_3_ clusters under radiation may have contributed to the improved dispersion, with the average cluster size decreasing to approximately 600–800 nm as shown in Fig. 10d′,f′. This enhanced distribution likely improves the composite’s gamma shielding effectiveness, as the high atomic number of tungsten enhances photon absorption for gamma radiation shielding.

**Fig. 11:**
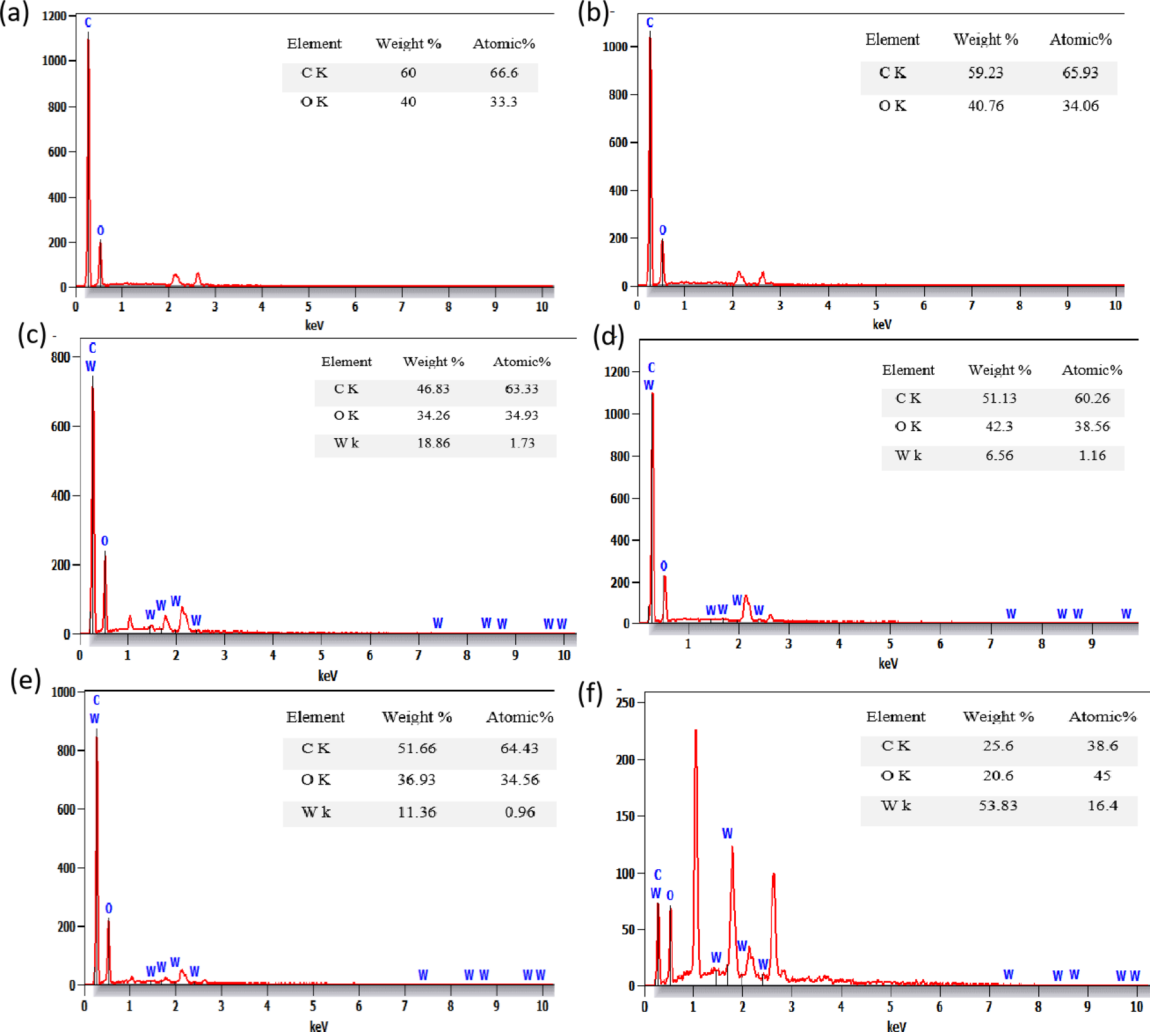
EDX image of the pure polymer PMMA (**a**, **b**), 40% (**c**, **d**), and 50% (**e**, **f**) of WO_3_ before and after radiation, respectively.

Identical imaging conditions were used to ensure that observed effects were due to irradiation. However, further mechanical tests are needed for a complete analysis. EDX analysis in Fig. 11a,b of pure PMMA sample before and after irradiation shows little change in the PMMA matrix due to gamma exposure. Figure 11c–f shows EDX analysis of PMMA with 40% and 50% WO_3_ before and after irradiation, indicating uneven carbon, oxygen, and tungsten distribution, which could impact gamma shielding performance. This could be due to the breaking and formation of chemical bonds under gamma radiation. The gamma radiation ionizes the polymeric chain, leading to chain crosslinking and scission via a free radical mechanism. The degree of crosslinking depends upon polymer structure, phase morphology, irradiation of gamma radiation controlled at a duration of 300 s, and nature of gamma radiation sources dose (point sources of Co-60, Cs-137, and Ba-133) that lead to the particle of tungsten oxide (WO_3_) has appeared clearly because of high loaded of 40%, and 50% WO_3_.

Figure 11e shows the EDX analysis of the PMMA/WO_3_ composite with 50% WO_3_ before irradiation, indicating weight percentages of carbon between 48.2 and 56.7%, oxygen between 34.8 and 40.7%, and tungsten between 2.5 and 16.5%. Atomic percentages range from 63.6 to 64.9% for carbon, 33.9% to 34.9% for oxygen, and 0.2% to 1.4% for tungsten, showing an uneven WO_3_ distribution that may impact gamma shielding performance. After irradiation, Fig. 11f reveals significant variations in composition: one site shows tungsten at 78.0% by weight with reduced carbon (8.4%) and oxygen (13.7%), while another site shows tungsten at 81.7% and minimal carbon (1.2%) and oxygen (17.1%). A third site displays tungsten at 1.8%, with oxygen at 31.0% and carbon at 67.2%. These differences reflect inconsistent distribution patterns, affecting the composite’s overall performance.

## Discussion

The PMMA/WO_3_ composite is effective at shielding against gamma radiation, which helps extend the life of satellites. Tests show that a concentration of 40 weight percent WO_3_ in PMMA provides the best balance for protecting against radiation while keeping the satellite safe. This concentration offers the most effective radiation shielding performance at gamma energies above 1000 keV and below 100 keV, it avoids issues like secondary emissions and agglomeration, which can reduce attenuation efficiency. WO_3_ at 50% concentration works best and is most effective for gamma radiation shielding in the energy range of 100–1000 keV. This is because, in comparison to other concentrations, it achieves the ideal and optimal balance between the amount of additive and the energy of the incoming gamma rays (absorption), enabling the material to maximize its radiation attenuation properties and offer superior protection.

The energy-dependent attenuation performance of PMMA/WO_3_ composites can be understood by considering the dominant photon interaction mechanisms. At higher energies, Compton scattering becomes the primary interaction mechanism, where WO_3_ provides superior attenuation due to its high atomic number and electron density. This trend aligns with previous studies on WO_3_ composites^7^, which have demonstrated enhanced shielding performance at higher photon energies. Given the relevance of high-energy gamma radiation in space environments—originating from galactic cosmic rays (GCRs) and secondary radiation—PMMA/WO_3_ composites offer a promising alternative for improving radiation shielding in microsatellite applications.

Improved mechanical strength, stiffness, and toughness are combined with increased thermal stability and flame retardancy in WO_3_/PMMA nanocomposites to create materials that are incredibly durable. A wide range of applications are made possible by their special qualities: WO_3_’s photocatalytic capability supports pollutant degradation for water and air purification; its high atomic number provides effective gamma radiation shielding for medical and industrial uses; its high gas sensitivity makes it easier to develop wearable gas sensors for environmental monitoring; and its electrochromic nature makes it possible to create flexible and transparent devices like smart windows, displays, and mirror.

Future studies can examine how various nanoparticle sizes and kinds affect these composites’ thermal performance and long-term thermal stability under cyclic thermal loading.

## Methods

In this study, WO_3_ nanoparticles were synthesized from tungsten powder, was obtained from Sigma-Aldrich, rather than using commercially available WO_3_. This approach was chosen to ensure better control over particle size, crystallinity, and purity, which are critical for achieving consistent and optimized performance in the PMMA/WO_3_ composite. Synthesizing WO_3_ in-house also allowed for customization of material properties to meet the specific requirements for radiation shielding and thermal stability in space applications. This nanoscale tungsten oxide was then mixed with PMMA, also provided by Sigma-Aldrich.

### Preparation

Poly methyl methacrylate (PMMA), Sodium tungstate (Na_2_WO_4_, purity ≥ 99%), and citric acid (C_6_H_8_O_7_, purity ≥ 99%) were obtained from Sigma-Aldrich, hydrochloric acid (HCl, Conc 37%), and chloroform (CHCl_3_) were obtained from Alpha Chemie.

To prepare the samples, 5 g of tungsten were dissolved in 20 millilitres of water and 10 g of citric acid. If the tungsten was not dissolved, 5 millilitres of HCL were added to dissolve it completely. Then, after pouring the dissolved solution into a large beaker, it was dried in a drying oven at 120 °C for 24 h, and then was placed in the muffle oven for two hours after the temperature reaches 550 °C. The product was grinded to turn it into a powder. After that, 20 g of PMMA were placed in a jar with 200 ml of chloroform, and stirred at 70 °C^8^. 50 millilitres of PMMA solution were then taken and differently prepared concentrations of metal nano oxide powder (0.0, 0.005, 0.05, 0.3, 20, 30, 40, 50) grams were added in this solution and were put in ultrasonication to be homogeny, then were put in ceramic plate or stainless and left to dry in room temperature. Lastly, it was ready to use it as composite^8^.

### Irradiation

**Fig. 12:**
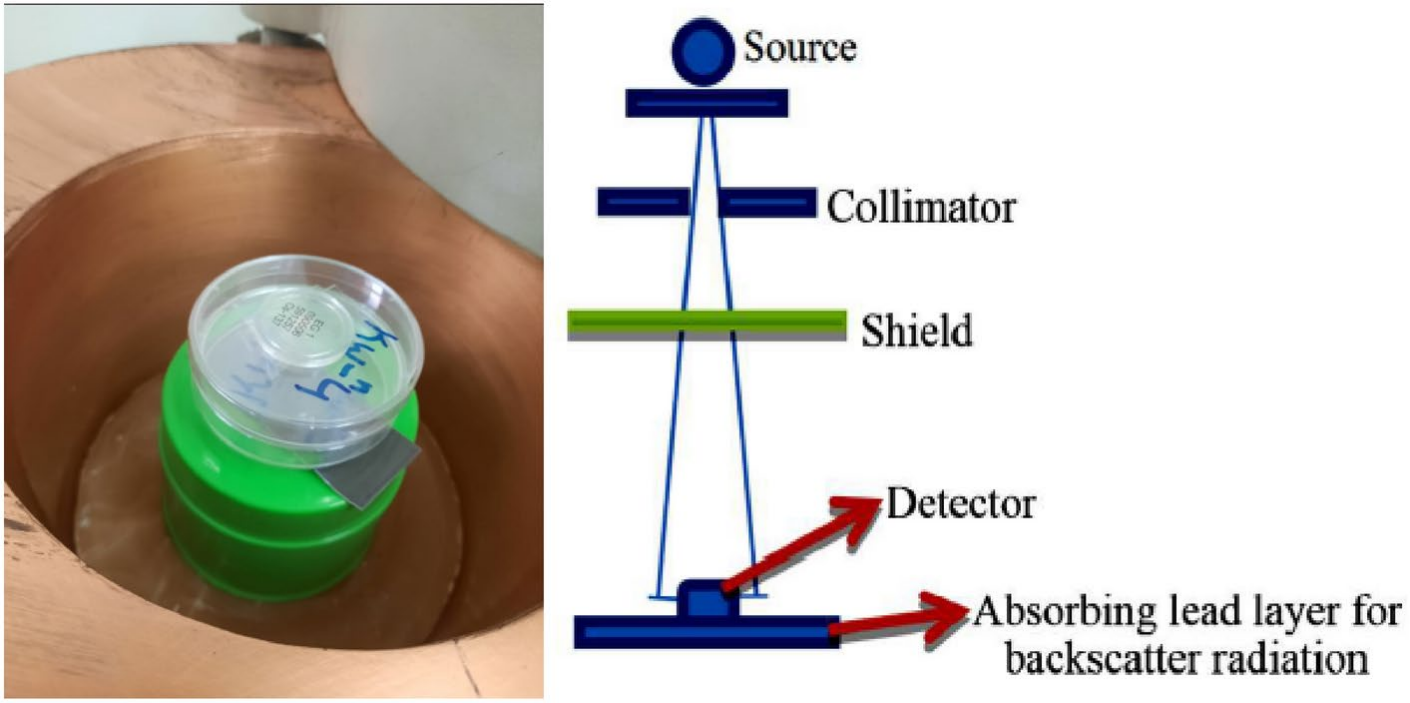
Gamma irradiation setup.

The radiation tests were carried out at The Egyptian Atomic Energy Authority (EAEA), Cairo, Egypt. The gamma shielding investigations were conducted using the apparatus shown in Fig. 12. The vertically oriented gamma radiation sources, Ba-133, Cs-137, and Co-60, emit gamma rays at energies of (79 and 356), (662), (1173 and 1332), keV respectively. The entire duration of the exposure was 300 s. The sources were contained in the shielding lead block, which was surrounded by lead blocks for partial collimation. The sample was positioned on a ring plate 1.5 cm from the source, and the NaI (Tl) detector with a base was positioned 8 cm from the sample’s base^1,11^.

Each sample undergoes five repetitions of the experimental measures.

Calculations were evaluated at different gamma energies for selecting the proper thickness of the gamma shielding from fabricated polymer nanocomposite^12^.

### Morphological characterization

A comprehensive understanding of PMMA/WO_3_ composites requires morphological characterization techniques such as X-ray Diffraction (XRD), Scanning Electron Microscopy (SEM) with Energy Dispersive X-ray (EDX) analysis, and Fourier-Transform Infrared (FTIR) spectroscopy. XRD (using the D8 DISCOVER model from BRUKER) provides insights into crystalline structure, phase composition, lattice strain, crystal size, and atom dislocation. High-resolution SEM (FEI Quanta FEG 250) is employed for surface and structural analysis, while ATR-FTIR spectroscopy (THERMO NICLOT, 50) identifies molecular interactions and functional groups. These techniques together offer detailed information on the micro and nanoscale structure, composition, and nanoparticle distribution within the PMMA matrix. They also help evaluate the interaction between WO_3_ particles and the polymer, as well as changes in structural and chemical properties. Such an in-depth analysis is critical for designing advanced materials with tailored properties for applications like radiation shielding. XRD analysis of PMMA/WO_3_ composites across various concentrations (0–50 wt%) revealed changes in phase composition and crystalline structure, affecting peak intensities and positions^8^. Paradoxically, the XRD spectra showed significant changes in peak forms and intensities upon exposure to radiation sources Ba-133, Cs-137, and Co-60, indicating that the crystalline structure of the composite was altered by radiation. Through a methodical analysis, the effects of radiation on various concentrations (0, 0.005, 0.05, 0.3, 20, 30, 40, and 50 wt%) were determined, offering insights into the structural resilience or vulnerability and ionizing radiation susceptibility. Regarding the PMMA/WO_3_ composite’s possible uses in radiation-sensitive settings, a thorough understanding of the effects of radiation were obtained^1,2^.

FTIR analysis applied to PMMA/WO_3_ composites to understand the molecular connections and structure inside the composite is known as material characterization. Ensuring the homogeneity and purity of the composite material is known as quality control. The research and development to examining how various manufacturing parameters or additives affect the characteristics of the composite. It takes careful examination and comparison with reference spectra of the individual components and established standards to comprehend the FTIR spectrum of a PMMA/WO_3_ composite^18^.

### Thermal characterization

Thermal analysis is crucial for understanding the behaviour of polymer composites under varying temperature conditions. Differential Scanning Calorimetry (DSC) and Differential Thermal Analysis (DTA) are powerful techniques used to study thermal transitions such as glass transition temperature (Tg) and melting point (Tm). This analysis is particularly important for PMMA/WO_3_ composites, which have potential applications in radiation shielding due to the unique properties imparted by WO_3_ nanoparticles.

DSC measures the heat flow into or out of a sample as it is heated, cooled, or held at constant temperature. This technique allows for the determination of temperatures and heat flows associated with thermal transitions in materials^19^.

Thermogravimetric analysis (TGA) provides crucial information on the composition and thermal stability of PMMA/WO_3_ composites, revealing that WO_3_ nanoparticles can enhance the composite’s thermal stability by raising the decomposition temperature (Td) and increasing residual mass. This study utilized the SETARAM instrument (France) with argon gas, operating at a maximum temperature of 1400 °C, a heating rate of 10 °C/min, and a cooling rate of 30 °C/min. Changes in the glass transition temperature (Tg) and melting point (Tm) with varying WO_3_ concentrations can indicate modifications to the polymer matrix’s stiffness, crystallinity, and nanoparticle interactions. Enhanced thermal stability or shifts in thermal transition temperatures suggest stronger interactions between PMMA and WO_3_. Understanding these thermal properties is essential for optimizing the use of PMMA/WO_3_ composites in high-temperature applications, such as radiation shielding, where thermal stability is crucial.

## Data Availability

The datasets generated and/or analyzed during the current study are available from the corresponding author upon reasonable request. All data pertain to experimental measurements and analyses conducted as part of this study.
